# The Ethical, Legal, and Social Issues Impacted by Modern Assisted Reproductive Technologies

**DOI:** 10.1155/2012/686253

**Published:** 2012-01-04

**Authors:** Paul R. Brezina, Yulian Zhao

**Affiliations:** Division of Reproductive Endocrinology and Infertility, Department of Gynecology and Obstetrics, Johns Hopkins Medical Institutions, Phipps 264 600 N. Wolfe Street, Baltimore, MD 21287, USA

## Abstract

*Background*. While assisted reproductive technology (ART), including *in vitro* fertilization has given hope to millions of couples suffering from infertility, it has also introduced countless ethical, legal, and social challenges. The objective of this paper is to identify the aspects of ART that are most relevant to present-day society and discuss the multiple ethical, legal, and social challenges inherent to this technology. *Scope of Review*. This paper evaluates some of the most visible and challenging topics in the field of ART and outlines the ethical, legal, and social challenges they introduce. *Major Conclusions*. ART has resulted in a tectonic shift in the way physicians and the general population perceive infertility and ethics. In the coming years, advancing technology is likely to exacerbate ethical, legal, and social concerns associated with ART. ART is directly challenging society to reevaluate the way in which human life, social justice and equality, and claims to genetic offspring are viewed. Furthermore, these issues will force legal systems to modify existing laws to accommodate the unique challenges created by ART. Society has a responsibility to ensure that the advances achieved through ART are implemented in a socially responsible manner.

## 1. Introduction

ART is currently a commonplace technology that has successfully treated millions of infertile couples the world over. However, the explosion of this technology has introduced a myriad of new social, ethical, and legal challenges. This paper evaluates some of the most visible and challenging topics in the field of ART and outlines the ethical, legal and social challenges they introduce.

## 2. Scope of ART Utilization

Infertility has traditionally been an area of medicine in which physicians had limited means to help their patients. The landscape of this field changed dramatically with the announcement of the birth of Louise Brown in 1978 through *in vitro *fertilization (IVF). This historic moment was eloquently encapsulated by Howard Jones who observed “Eleven forty-seven p.m. Tuesday, July 25, 1978, was surely a unique moment in the life of Patrick Steptoe. This was the hour and minute he delivered Louise Brown, the world's first baby, meticulously, lovingly, and aseptically conceived in the laboratory, but popularly referred to as the world's first test tube baby” [1]. The importance of this birth to scientists, clinicians, and most particularly infertile patients throughout the world cannot be overstated. In several short decades, IVF has exploded in availability and use throughout the world.

Worldwide, more than 70 million couples are afflicted with infertility [2]. Since the first successful IVF procedure in 1978 [3], the use of this and related technologies has expanded to become commonplace around the globe. Over the past decade, the use of ART services has increased at a rate of 5–10% annually [4, 5].

In 1996, approximately 60,000 IVF cycles were initiated in the United States with approximately 17,000 clinical pregnancies and 14,000 live births [6]. Currently, IVF accounts for approximately 1% of all live births in the United States [6]. As of 2009, 3.4 million children have been born worldwide after ART treatment, and ART utilization is currently increasing at a rate of 5–10% annually in developed countries [4].

## 3. Reporting Regulations

The widespread use of this technology throughout the world has prompted a desire by the public, governmental bodies, and professional organizations to create mechanisms that evaluate the utilization of ART. Advances in the arena of assisted reproductive technologies (ART) are accompanied by ethical and societal concerns. Legislation and professional societies have attempted to address these concerns for some time. For example, in 1986, the American Fertility Society first published guidelines for the ethical implementation of ART in the United States [7]. The dynamic nature of ART and the rapid evolution of the field result in constant paradigm shifts that require frequent and comprehensive evaluation by professional organizations and society alike.

In the 1980's, concerns surrounding ART focused on the safe administration of gonadotropins, transparency of pregnancy data from clinics, and addressing economic barriers to ART access. Some of these issues, such as reporting requirements for ART pregnancy results, have also been mandated with legislation in many nations [8]. Furthermore, ART reporting requirements generally include the number of embryos transferred. This measure has been extremely important in correlating the risk of multiple gestations with the transfer of 2 or more embryos. However, in many nations, reporting regulations are not accompanied by legislation defining practice patterns. For example, in the United States, while physicians are required to report the number of embryos transferred in an IVF cycle, there are no laws that state the allowed number of embryos transferred [8].

Through centralized mandatory reporting registries, general estimates of IVF activity are available in many nations. In an effort to define current IVF statistics and to make this information more transparent and available to patients, the Fertility Clinic Success Rate and Certification Act of 1992 was created in the United States [8]. This law requires clinics providing IVF in the United States to report specific information regarding IVF cycles, including pregnancy rates [6]. This reporting data is only reported on IVF cycle outcomes and does not include detailed information regarding the maternal or paternal medical history [6]. In other countries, similar national registries exist [5], making it possible to evaluate data from IVF cycles on both a national and international scale. A detailed accounting for ART reporting and regulations across the globe is available from the International Federation of Fertility Societies (IFFS) [5]. In their 2010 report, the IFFS reported ART outcomes data from 59 countries [5].

Such laws were implemented in an attempt to ensure that patients may be informed as to which clinics have superior ART pregnancy results. In some instances, however, this has led to some clinics “cherry picking” patients to improve their overall pregnancy results. This has actually become a barrier to receiving ART for many patients with a relatively poor pregnancy prognosis.

## 4. Practice Regulations and Multiple Gestation Pregnancies

Federally mandated regulations, however, are not limited to registries. Increasingly, nations have enacted legislation that defines the parameters for acceptable practice of ART. The transfer of multiple embryos in a single cycle increases the rates of multiple births [9]. Because of the increased social costs and health risks associated with multiple births, legislation or guidelines from professional societies have been introduced in many countries restricting the number of embryos that may be transferred per IVF cycle in an effort to limit the incidence of multiple gestations [9–11]. Indeed, a study in the United Kingdom found that the total health care system costs following a singleton birth were *£*3313, *£*9122 following a twin birth and *£*32,354 following a triplet birth [9]. Additionally, the health risks, both to the mother and the infant, increase dramatically with increasing number of infants [9]. In the United States in 2007, the number of embryos transferred per cycle ranged from 2.2 in women under 35 to 3.1 in women over 40 years of age (CDC). Multiple birth rates in the United States in 2007 ranged from approximately 35% in women under 35 to 15% in women over the age of 40 [12]. In Europe, the approximate number of embryos transferred in the year 2006 was one (22%), two (57%), three (19%), or four (1.6%) [13]. In 2007, 79.2% of European births were singletons, with a twin rate of 19.9% and a triplet rate of 0.9% [5].

Pregnancy rates associated with IVF are high compared to those seen in the early days of the procedure. The current efficiency of IVF is more cost effective and efficacious in achieving pregnancy than other modalities, such as injectable gonadotropins coupled with intra uterine insemination (IUI), which traditionally some have preferred [14]. The increased efficiency of IVF has also resulted in an increased rate of multiple gestations. Recent data suggests that single embryo transfer, coupled with subsequent frozen embryo transfer, results in equivalent pregnancy rates compared with the transfer of multiple embryos, without an increase in multiple pregnancy rates [11]. Additionally, single embryo transfer would inherently decrease maternal and infant health risks associated with multiple gestation pregnancies [9]. Therefore, a trend toward single embryo transfer is likely to increase in the future.

Variability of legislation regulating IVF exists in different countries and even states/provinces within a single nation [6]. For example, in an effort to minimize multiple gestation pregnancies resulting from ART, some laws place limits on the number of embryos that may be transferred, cryopreserved, or fertilized per IVF cycle [5, 6, 15, 16]. In some cases, these regulations or fiscal pressures result in couples traveling across international border to obtain treatments that are unavailable in their native country [17]. This practice, known as cross-border reproductive care (CBRC), is thought to account for as much as 10% of the total IVF cycles performed worldwide [17, 18].

## 5. Financial Aspect for IVF Treatment

Perhaps one of the most obvious ethical challenges surrounding ART is the inequitable distribution of access to care. The fact that significant economic barriers to IVF exist in many countries results in the preferential availability of these technologies to couples in a position of financial strength [19]. The cost of performing ART per live birth varies among countries [4]. The average cost per IVF cycle in the United States is USD 9,266 [20]. However, the cost per live birth for autologous ART treatment cycles in the United States, Canada, and the United Kingdom ranged from approximately USD 33,000 to 41,000 compared to USD 24,000 to 25,000 in Scandinavia, Japan, and Australia [14]. The total ART treatment costs as a percentage of total healthcare expenditures in 2003 were 0.06% in the United States, 0.09% in Japan, and 0.25% in Australia [4]. Some have maintained that the cost for these cycles pales in comparison to the social advantages yielded by the addition of productive members of society [21]. This is especially true in societies that have a negative or flat population growth rate coupled with an aging population [21].

The funding structure for IVF/ART is highly variable among different nations. For example, no federal government reimbursement exists for IVF in the United States, although certain states have insurance mandates for ART [4, 19, 22]. Many other countries provide full or partial coverage through governmental insurance [4, 9]. In many instances, long waiting times for IVF through these government programs encourage couples to seek treatment in private fertility centers that accept remuneration directly from the patients [4, 23, 24]. In the United Kingdom, for example, only approximately 25% of all IVF cycles performed are funded by the National Health Service [9].

## 6. Preimplantation Genetic Testing

Preimplantation genetic screening (PGS) and diagnosis (PGD) offer the unique ability to characterize the genetic composition of embryos prior to embryo transfer. Given the recent successes of these technologies, the broader implementation of this technology in the future is likely. Although controversial, using PGD to choose embryos solely on the basis of gender is currently being practiced [25, 26]. Sex selection in the proper setting may offer a substantial health benefit. For example, choosing to transfer only embryos of a certain sex may confer a therapeutic benefit if used to avoid a known sex linked disorder. However, sex selection PGD purely for the preference of the parents could conceivably, if practiced on a large scale, skew the gender proportions in certain nations where one gender is culturally preferred.

In the near future, with refinements in microarray technology and the defining of genetic sequences associated with certain physical characteristics, it is conceivable that specific physical or mental characteristics may be evaluated to guide the decision as to which embryos to transfer. This possibility raises concerns on both ethical and practical levels. Of more concern is the possibility that in the future, technology will permit the manipulation of genetic material within an embryo. Rigorous public and scientific oversight of these technologies is vital to ensure that scientific advances are tempered with the best interests of society in mind.

## 7. Fertility Preservation

Female fertility is well documented to decrease with age [27, 28]. Consequently, much research has been conducted aimed at preserving female fertility before advanced age is realized. Additionally, fertility preservation for individuals afflicted with cancer has important implications as often the chemotherapeutic agents used to treat cancer are toxic to the ovary and result in diminished ovarian reserve and reduced fertility. While techniques for freezing sperm and embryos are well established, techniques for freezing oocytes and ovarian tissue are still considered experimental [29]. Multiple techniques including oocyte cryopreservation and preservation of strips of ovarian cortex with subsequent reimplantation and stimulation have been described, with some pregnancy success [30–33]. Fertility preservation for cancer patients using *in vitro *maturation (IVM), oocyte vitrification and the freezing of intact human ovaries with their vascular pedicles have also been reported [34]. As of 2008, more than 5 babies had been delivered through IVF following ovarian tissue transplantation [35]. Many have suggested that, prior to being treated for cancer, women should be offered fertility preservation measures as outlined above [34].

Recently, several laboratories have demonstrated the ability to successfully cryopreserve oocytes following an IVF cycle. These developments have profound implications. As the birth control pill gave women the ability to prevent pregnancy, oocyte cryopreservation may give women the flexibility to preserve their fertility potential, starting at a young age, while postponing childbearing. However, as this technology at the present time in many countries is generally only available to those with financial means. This poses ethical and social issues that will certainly see more attention in the future.

## 8. Gamete Donation

The use of donor gametes, either in the form of donor sperm or donor oocytes, is commonplace in ART. The use of donor sperm can be traced to the 1800's [36]. In the mid 1980s, oocyte donation was introduced [36]. In recent years, issues surrounding the use of donor gametes have become increasingly visible [37]. Women donating oocytes must undergo IVF. Due to the inherent medical risks associated with IVF, including ovarian hyperstimulation syndrome and surgical risks, a central concern of allowing women to be oocyte donors includes adequate informed consent [37]. Consent, in addition to outlining these medical risks, should include counseling regarding the emotional benefits and risks of donation with an emphasis that long-term data regarding these risks are lacking [37]. Additionally, it is considered an ethical prerequisite that oocyte donors participate voluntarily and without coercion or undue influence [38]. Some have expressed concern that financial compensation of oocyte donors may lead to exploitation as women may proceed with oocyte donation against their own best interests, given the inherent medical risks involved [39]. The concept of commodification, that any “buying or selling” of human gametes is inherently immoral, is an additional argument used against remunerating women serving as oocyte donors [39]. Due to the substantial controversy surrounding oocyte donation, especially the amount of financial compensation may be given to an oocyte donor, federal regulations governing this practice are constantly evolving and differ substantially from country to country [39].

Another ethical and legal issue surrounding the use of donated gametes is to what extent the anonymity of the donor should be preserved. The issue of anonymity as it relates to gamete and embryo donation is emotionally charged. Indeed, the ability of human beings to know their genetic roots is universally important, at the core of self identity. Either egg and sperm donors may choose to or not to be anonymous, though the vast majority in both groups generally chooses anonymity [40]. The American Society for Reproductive Medicine has identified four levels of gamete donor information sharing depending on the wishes of the donor and recipient parties [37]. Recently, however, there is, increasing consideration of the rights of offspring as it relates to donor gametes and anonymity [40]. Advocates for allowing either gamete donors or their offspring to break anonymity cite the medical advantages of sharing medical information with their genetic offspring, in the case of the donor, or learning about their genetic history directly, in the case of offspring [41, 42]. Others simply argue that both donors and offspring have an inherent right to meet and develop a relationship [43]. Recent court rulings suggest that these rights will become more visible in the future. For example, in the British case *Rose v Secretary of State for Health [2002] EWHC 1593*, the court ruled that based on the Human Rights Act, donor offspring could obtain information about their genetic parents despite previously established anonymity [43]. The ethical and legal issues surrounding anonymity and gamete donation are sure to be a centrally debated issues within the field of ART for the foreseeable future.

## 9. Embryo Donation

IVF cycles often result in couples transferring several embryos and cryopreserving other embryos produced by the cycle, presumptively for the purpose future pregnancy. However, in many instances, these surplus embryos are never used by the genetic parents and therefore are stored indefinitely [44]. The number of such embryos stored internationally is surprisingly high. In the United States alone, it is estimated that over 400,000 embryos are currently cryopreserved, many of which will not be used by their genetic parents [44]. The ethical and moral issues surrounding how to deal with these surplus embryos have been the source of much debate. In general, four possible fates for these embryos exist [44]:

1. thawing and discarding,
2. donating to research,
3. indefinite storage,
4. donating the embryos to another couple for the purposes of uterine transfer.

All of these strategies have staunch supporters and detractors. Not surprisingly, there are a myriad of laws in different countries governing many aspects of how a human embryo that has been cryopreserved may be handled [44, 45]. The use of embryos for the purpose of research, specifically as it relates to human stem cells, has also been a source of fierce debate internationally and has resulted in substantial regulation that varies substantially from nation to nation [46–49].

## 10. Surrogacy and Gestational Carriers

Another topic of ethical, social, and legal debate surrounds the use of surrogacy and gestational carriers. Surrogacy is defined as a woman who agrees to carry a pregnancy using her own oocytes but the sperm of another couple and relinquish the child to this couple upon delivery [50]. A gestational carrier, by contrast, involves a couple who undergoes IVF with their genetic gametes and then places the resultant embryo in another woman's uterus, the gestational carrier, who will carry the pregnancy and relinquish the child to this couple upon delivery [50]. Currently, the use of gestational carriers is far more common than that of surrogates [50].

As with donor gametes, surrogates and gestational carriers are subject to significant medical and emotional risks from carrying a pregnancy and undergoing a delivery [50]. As such, extensive counseling and meticulous informed consent are required [50]. Some also are concerned that the use of surrogates and gestational carriers is a form of “child selling” or the “sale of parental rights” [51]. Additionally, the rights of the surrogate or gestational carrier to not relinquish the infant following deliver are not well described [50]. In fact, legal precedent in some states within the United States has actually upheld the right of a birth mother, regardless of genetic relation to the child, to retain parental rights despite the existence of a preexisting gestational carrier contract [50].

Another central concern surrounding the use of surrogates and gestational carriers is the possibility that financial pressures could lead to exploitation and commoidification of the service [50–53]. The mean compensation for a gestational carrier in the United State in 2008 was estimated at approximately $20,000 [50]. In contrast, a gestational carrier in India receives an average of $4,000 for the same service [52]. Regulation of surrogates and gestational carriers varies widely from nation to nation and even within regions of individual countries [50, 52–56]. Due to these financial and legal considerations, international surrogacy has emerged as an emerging industry, especially in developing nations [52]. This practice has exacerbated the already difficult ethical and legal issues surrounding gestational carriers [52]. At the present time, issues surrounding issues of individual rights, commoidification, exploitation, citizenship of the offspring of international gestational carriers, and even fair trade are largely unresolved internationally [52, 55].

## 11. Possible Deleterious Effects of ART

There are questions that remain outstanding regarding the use of IVF. Conflicting data exists about the risks of IVF on the developing embryo. Multiple studies have failed to find a clinically relevant association between IVF or embryo cryopreservation and adverse maternal or fetal effects [57–59]. Other studies have suggested that infants of IVF pregnancies may be at a small but statistically significant increased risk for rare epigenetic and other abnormalities [60–62].

Despite this controversy, there is a general consensus that IVF confers a small but measurable increased risk for a variety of congenital abnormalities including anatomic abnormalities and imprinting errors as compared to the general population [63]. Some maintain, however, that this is secondary to an increased baseline risk for these problems in the population of infertile patients [63]. Regardless of the cause, this small increased risk, while statistically significant with extremely large sample sizes, will likely not be a powerful enough factor to dissuade infertile couples from pursuing parenthood through IVF.

## 12. Conclusion

ART has emerged as one of the most widely adopted and successful medical technologies in the last century. While giving hope to millions of couples suffering from infertility, ART also has presented new ethical, legal, and social questions that society must address. Many countries have taken steps to regulate certain aspects of ART. Specifically, what regulations and laws should be in place for ART reporting, social inequities that may arise from financial barriers to ART, genetic testing, emerging laboratory techniques that have improved embryo and gamete survival when cryopreserved, and an individual's right to their genetic offspring in the setting of gamete or embryo donation are aspects of ART which will become increasingly controversial and debated into the future.

However, the lion's share of ethical and legal questions that exist surrounding ART have yet to be resolved. Society must reconcile how to fund ART in a responsible and equitable manner to increase access to care. Additionally, the myriad of unresolved issues surrounding gamete and embryo donation must be addressed in greater detail in future social and legal dialogues.

ART is a field that is dynamic and ever changing. In areas of ART such as preimplantation genetics, new technologies continually change the capabilities of ART. Due to the rapidly evolving nature of the ART, legislation is often unable to keep pace and address all of the ethical and legal issues that are constantly emerging in the field. It is therefore incumbent upon physicians to continually monitor these issues and ensure that ART technologies are offered and delivered in a manner that balances patient care with social and moral responsibility.
